# The Protein Histidine Methyltransferase METTL9—From Mechanism to Biological Function

**DOI:** 10.3390/life16030445

**Published:** 2026-03-09

**Authors:** Pål Ø. Falnes, Erna Davydova

**Affiliations:** 1 Department of Biosciences, Faculty of Mathematics and Natural Sciences, University of Oslo, 0316 Oslo, Norway; 2 CRESCO—Centre for Embryology and Healthy Development, University of Oslo and Oslo University Hospital, 0373 Oslo, Norway

**Keywords:** methyltransferase, protein methylation, METTL9, histidine methylation, zinc, *S*-adenosylmethionine

## Abstract

Proteins can be methylated at either of the two N atoms of the imidazole ring of histidine, yielding 1-methylhistidine (or pi-methylhistidine) or 3-methylhistidine (tau-methylhistidine). While protein histidine methylation in mammals was discovered more than 50 years ago, the first histidine methyltransferases were identified only recently. So far, four different human protein histidine methyltransferases have been uncovered, and one of these is METTL9, which is responsible for introducing 1-methylhistidine in a number of proteins. The minimal sequence motif that is required, though not always sufficient, for METTL9-mediated methylation is His-X-His (HxH), where X is preferentially a small uncharged residue. Many METTL9 substrates are methylated at stretches of alternating histidines, i.e., several adjoining HxH motifs, such as HxHxH. Histidines are frequently involved in binding metal ions, such as zinc. Accordingly, it has been shown for several sequences targeted by METTL9, for example, in the immunomodulatory and antibacterial protein S100A9 and the zinc transporter SLC39A7, that histidine methylation diminishes zinc binding and thereby modulates protein function. In this review, we present a detailed account of METTL9-mediated histidine methylation, regarding its discovery, biochemical mechanism, structural features, and biological significance.

## 1. Introduction

**Figure 1:**
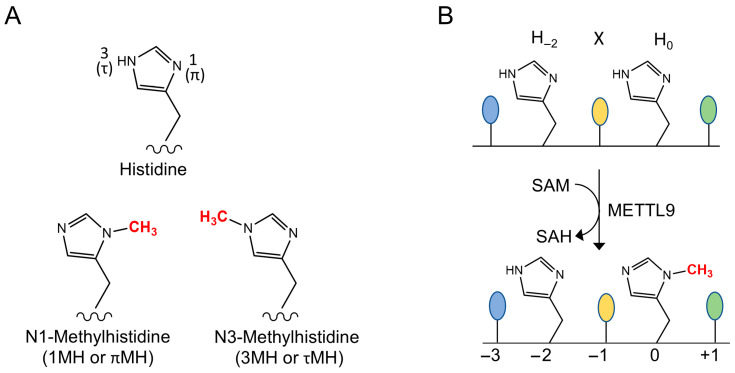
The METTL9 reaction. (**A**) The two isomers of N-methylated histidine. (**B**) The reaction catalyzed by METTL9. A methyl group is transferred from *S*-adenosylmethionine (SAM) to the N1-atom of the second histidine residue (denoted H_0_) of the HxH motif, yielding *S*-adenosylhomocysteine (SAH) as a byproduct. The first residue of the HxH motif (H_−2_) remains unmethylated. A small residue at the x (−1) position is preferred for efficient methylation.

Histidine residues in proteins can be methylated at either of the two N atoms of the imidazole ring, generating 1-methylhistidine (1MH; aka π/“pi”-MH) or 3-methylhistidine (3MH; aka τ/“tau”-MH) (Figure 1A). This was discovered more than 50 years ago, when it was reported that actin and myosin from striated muscle contain 3MH and 1MH, respectively, as observed in various vertebrates, including rabbits, chicken, and fish [1,2,3]. However, the scientific progress on protein histidine methylation was limited during the ensuing decades, with only a handful of additional histidine-methylated proteins identified in mammals. These were myosin light-chain kinase (MYLK2) from rabbit skeletal muscle [4], the mitochondrial Complex I component NDUFB3 from bovine heart [5], and the anti-inflammatory and immunomodulatory protein S100A9 from mouse spleen [6]. Also, the responsible methyltransferases (MTases) remained elusive, thus making it difficult to assess the biological significance of these methylation events.

However, during the last few years, it has become clear that histidine methylation is more pervasive than previously thought, and a number of important discoveries were made. In 2018, the Gozani and Drozak groups independently reported that 3MH in actin is installed by the MTase SETD3 and is important for actin and smooth muscle function [7,8]. Subsequently, we and others uncovered the hitherto uncharacterized MTase METTL9, the topic of the present review, as the first 1MH-generating protein MTase [9,10,11]. It was also shown that METTL18 is responsible for introducing a single, functionally important 3MH modification in the ribosomal protein RPL3 [12,13], a biochemical activity that had already been assigned to its yeast ortholog Hpm1 [14]. More recently, it was shown by the Shinkai group that CARNMT1, initially found to introduce 1MH in the unconventional dipeptide carnosine but also predicted as a protein histidine MTase [15], targets histidines in so-called C3H zinc fingers [16]. This finding was subsequently confirmed by two independent studies [17,18].

Almost all of the ~200 human methyltransferases use *S*-adenosylmethionine (SAM) as their methyl donor, and, based on their sequence and structure, they can be subdivided into several distinct families [19,20]. The largest of these is the so-called seven-β-strand (7BS) family, which encompasses ~120 MTases in humans [21]. The second largest is the SET-domain family, which comprises ~55 human MTases that primarily methylate lysines [22]. Interestingly, the first discovered human protein histidine MTase SETD3 also belongs to this family. All three other human histidine MTases (METTL9, METTL18, and CARNMT1) are 7BS MTases.

The 7BS MTases share a seven-stranded twisted beta sheet of a distinct topology, where residues involved in binding the co-substrate are found as sequence motifs at distinct locations in the structure. Of these, motif “I”, typically with the sequence GxGxG, and “Post I”, which contains a critical acidic (Asp/Glu) residue, are involved in binding the cofactor SAM [23,24]. Motif “Post II”, which contains residues interacting with the substrate to be methylated, shows only a limited sequence similarity across the entire 7BS MTase family, but is highly conserved between MTase orthologs, and within subfamilies of MTases that target similar substrates [23,24].

METTL9 is a 7BS MTase that introduces 1MH in several human proteins, and putative orthologs are found in a wide range of multicellular and unicellular eukaryotes. METTL9 specifically targets the sequence His-x-His (HxH) (Figure 1B), and “x” is typically a small uncharged amino acid. As only the second histidine becomes methylated, it will here be defined as “position zero” and referred to as H_0_. Thus, the first histidine of the HxH motif will be denoted H_−2_, with other positions numbered accordingly. In the present review, we describe the discovery of METTL9 as a histidine-specific MTase, and we also discuss METTL9’s specificity and substrate recognition on the backdrop of the recently published METTL9 structures. Finally, we will elaborate on the biological significance and disease relevance of METTL9 and also review recent findings on a potentially important function for a circular RNA derived from the METTL9 gene.

## 2. Discovery of METTL9 as a Histidine Methyltransferase

The biochemical function of METTL9 was uncovered in three independent studies, all published in 2021 [9,10,11]. We, in collaboration with the Shinkai and Jeltsch groups, investigated METTL9 as a potential protein MTase by initially assessing its ability to methylate proteins in a cell extract [10]. We used [^3^H]SAM as a methyl donor, thus enabling the detection of methylated proteins as radiolabeled bands when proteins are separated by gel electrophoresis. Indeed, several labeled bands were detected, and, importantly, many of these were considerably stronger when extracts from METTL9 knock-out (KO) cells were used compared to wild-type extracts [10]. This indicated that these bands represent bona fide METTL9 substrates that are close to fully methylated in the WT cells, but unmethylated in the KO cells. Combining this approach with protein mass spectrometry (MS), we were able to identify ARMC6 as a METTL9 substrate, and we found that the sequence HAHNH was histidine-methylated [10]. Interestingly, similar HxHxH sequences had previously been reported to contain methylhistidine in other mammalian proteins, i.e., murine S100A9 and bovine NDUFB3. Moreover, we noted that zinc transporter proteins of the SLC39A (ZIP) and SLC30A (ZNT) families, which were identified as METTL9 interactants [25], are extremely rich in tracts of alternating histidines. Indeed, we found that peptide substrates encompassing HxHxH tracts from S100A9, ARMC6, and the zinc transporter SLC39A5 were all methylated by METTL9, and we identified HxH as the minimum motif required for methylation [10]. Furthermore, using three different HxH-containing peptides, and varying the middle residue x, we found that the small residues alanine, asparagine, glycine, serine, and threonine (A, N, G, S, T) were preferred at this position, and we dubbed the METTL9 recognition sequence as an “ANGST HXH motif” [10]. We also used an assay based on proteomics and the clickable SAM analog ProSeAM to identify additional candidate METTL9 substrates in cell extracts, and several of these were verified using peptides or recombinant proteins [10]. For six of the in vitro substrates of METTL9, in vivo methylation was also demonstrated [10]. These encompassed the previously mentioned ARMC6, NDUFB3, and S100A9, as well as the zinc transporter SLC39A7, the chaperone DNAJB12, and cyclin T2 (CCNT2), but, for the latter protein, in vitro and in vivo methylation was observed on different sequences [10].

In a second study, Lv and coworkers studied METTL9 in the context of tumor growth, but also investigated its biochemical activity [11]. They found that recombinant METTL9 methylated a ~55 kDa protein in cell extracts and then tested as a candidate substrate the zinc transporter SLC39A7, noting that it is a METTL9 interactant of a matching molecular weight [11]. Indeed, they found that several HxH containing fragments from SLC39A7 could be methylated by METTL9. Similar to our work, they found that HxH was the minimal sequence required for methylation, and that efficient methylation required one of the small residues, A, C, G or S, in the x position [11] (note that we, due to technical limitations, did not include C when examining the x residue).

A third study, by Daitoku et al., focused on identifying the elusive MTase responsible for histidine methylation of S100A9 [9]. To this end, they used siRNAs to knock down a panel of candidate MTases in mouse cells ectopically expressing FLAG-tagged S100A9. They then determined the 1MH content of immunoprecipitated S100A9 through analyzing its constituent amino acids using LC-MS/MS [9]. Only in the case of METTL9 did siRNA-mediated knockdown lead to a substantial reduction in 1MH content, suggesting METTL9 as the MTase responsible for S100A9 methylation [9]. Indeed, recombinant METTL9 was found to methylate S100A9 in vitro, and the overexpression of METTL9 dramatically increased the 1MH content of S100A9 in cells [9].

## 3. METTL9—Substrate Recognition and Enzyme Processivity

While an ANGST HxH motif is required for efficient METTL9-mediated methylation, it is not always sufficient. In part, this became evident when we investigated a set of 56 HxH-containing candidate substrate peptides for METTL9-mediated methylation in vitro [10]. These mainly represented HxH sequences found to be methylated in cells and HxH motifs in METTL9-interactants. We observed, even when considering only the peptides with an optimal motif (regarding the −3, −1 and +1 positions), that the degree of methylation varied greatly, even with some of the peptides showing no detectable methylation [10]. Thus, additional sequence and structural features, apart from the identity of the −3, −1 and +1 residues, also appear to play a strong role. Moreover, for one HxH-containing sequence, namely from NDUFB3, no in vitro methylation was observed on a short peptide, but the sequence was efficiently methylated by METTL9 in the context of both the recombinant and cellular NDUFB3 proteins [10]. This may suggest that, for some substrate proteins, regions beyond the immediate vicinity of the methylation site contribute to METTL9 binding, and/or that the structuring of the target sequence may somehow promote methylation in the folded protein.

**Figure 2:**
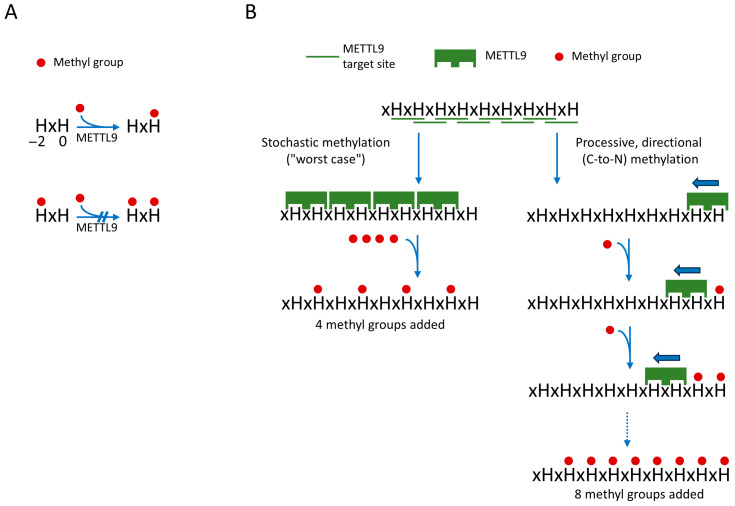
Processive directional action of METTL9 on tandem HxH tracts will yield increased methylation. (**A**) Unmethylated H_−2_ is required for METTL9-mediated methylation of H_0_. (**B**) Comparison of a processive versus stochastic mode of METTL9 action. A processive mode of action (right), where METTL9 methylates consecutive HxHs in the C-to-N direction, which will allow for methylation of all HxHs. In contrast, a stochastic action will frequently lead to the destruction of a neighboring (C-proximal) site, since methylation of H_−2_ of the HxH motif will prevent methylation of the H_0_ residue. This is illustrated by the hypothetical example of a substrate with nine alternating histidines, i.e., eight distinct HxH motifs. Here, a C-to-N fully processive mode will yield methylation of eight the histidines, whereas a stochastic mode, in the worst-case scenario, will only produce four methylations. The figure is inspired by figure 7 in Zhao et al. [27].

In our initial publication describing the activity of METTL9, we found no cases of methylation at the first histidine residue (H_−2_) of the HxH motif (or of tandem HxHs, such as HxHxH), and we therefore suggested that only the second histidine (at H_0_) of a HxH motif is a target of methylation [10]. Indeed, this was later strongly supported by structures of complexes between METTL9 and HxH-containing peptide substrates (see also the next section) [26,27]. Moreover, it was shown by Zhao et al. that an HxH-containing synthetic peptide with an N1-methylated first histidine was not subject to further in vitro methylation by METTL9 [27], as also illustrated in Figure 2A. Thus, in stretches of tandem HxHs, the methylation of an HxH will block the methylation of the downstream, partially overlapping HxH. Consequently, methylation of all sites within a tandem HxH stretch can be achieved only if METTL9 starts at the most C-proximal site and then in a processive manner methylates the upstream sites consecutively in a C-to-N direction, as illustrated in Figure 2B. In contrast, a stochastic, non-processive mode of methylation will destroy potential METTL9 target sites and give a lower level of methylation (Figure 2B). Zhao et al. further investigated the processivity of METTL9-mediated methylation using a substrate containing seven alternating histidine residues (i.e., six HxHs) and then studied the methylation pattern using MS [27]. Interestingly, a clear tendency for the clustering of methylations at adjoining HxH motifs was observed, indicating that METTL9 acts in a processive manner [27]. This may be beneficial for obtaining high methylation levels in long tandem HxHs, found, e.g., in human zinc transporters. However, even though METTL9 seems to be a processive enzyme, a complete methylation of long stretches of tandem HxHs would likely occur rarely, as this would depend on METTL9 entering at the last (most C-proximal) HxH of the HxH tract. Also, it will be interesting to investigate whether the apparently processive nature of METTL9 is due to a sliding mechanism, i.e., that the enzyme, after having catalyzed methylation, stays associated with the protein substrate and slides to the next HxH, or if METTL9 dissociates from the substrate between methylation events, and that the apparent processivity merely is due to a propensity of the enzyme to re-enter on a neighboring upstream HxH.

## 4. The Structure of Human METTL9

Two independent studies from 2023, by Wang et al. and Zhao et al., solved the structure of human METTL9 using X-ray crystallography, presenting structures of METTL9 in complex with *S*-adenosylhomocysteine (SAH) and various HxH-containing substrate peptides [26,27]. Both studies reported structures of the core, enzymatically active MTase domain of METTL9, lacking the N-terminal portions of the 318 aa protein. Zhao et al. solved, at a 2.5 Å resolution, the structure of METTL9_37–318_ in complex with SAH, and also presented a 3.4 Å structure of METTL9_37–318_ in complex with both SAH and an SLC39A7-derived substrate peptide [27]. Similarly, Wang et al. presented several structures, in the resolution range 1.69–3.43 Å, of METTL9_46–318_ in complex with SAH and peptides derived from SLC39A5 or S100A9 [26]. In order to increase solubility and homogeneity, this study used a METTL9 mutant, where six surface-exposed hydrophobic residues had been replaced.

**Figure 3:**
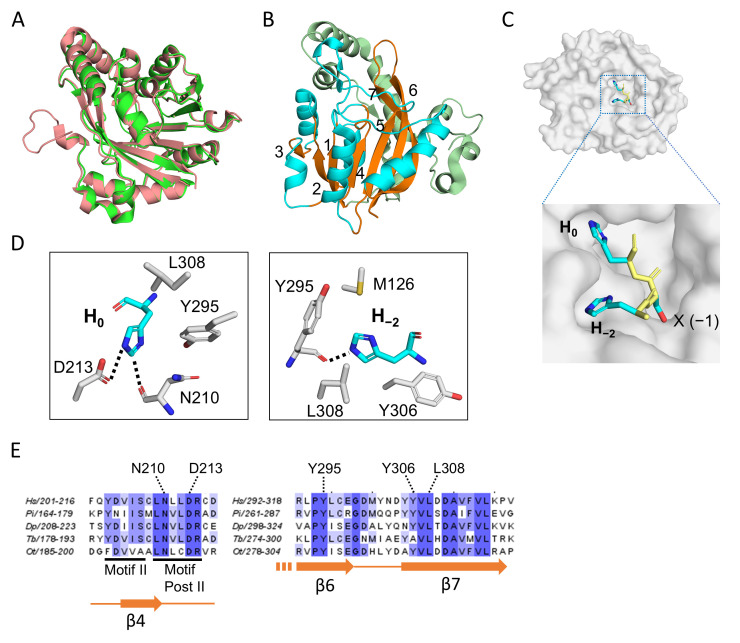
The structure of human METTL9. (**A**) Superposition of human METTL9 structures from the two X-ray crystallography studies [26,27]. In green is shown the 3.4 Å structure obtained by Zhao et al. (pdb 8gze; amino acids 55–317), which was obtained in complex with a SLC39A7-derived peptide [27]. The 1.69 Å structure from Wang et al. (pdb 7yf2) is shown in salmon color and was obtained as a complex with an SLC39A5-derived peptide [26]. This structure encompasses residues 44–318 and was generated from a mutant form of METTL9, where six hydrophobic surface-exposed residues had been substituted to increase solubility. In both cases, SAH was also part of the structure. (**B**) The overall structure of human METTL9. The seven-stranded β-sheet is shown in orange with the individual strands numbered. The α-helices located between the β-strands (in the sequence) are in cyan, and the segment N-terminal of the β-sheet is shown in pale green. (**C**) The HxH-binding cavity of METTL9. The structure of the HxH portion of the SLC39A5-peptide is shown (as sticks) on a surface representation of METTL9 (in gray). The amino acid side chains (x = S) are shown in cyan, while the main chain is shown in yellow. (**D**) Key METTL9 residues interacting with the invariant H_0_ (**left**) and H_−2_ (**right**) of the HxH-motif. The structure presentations (in **A**–**D**) were all made using the PyMol Molecular Graphics System (Schrodinger, LCC, ver. 3.1.6.1), and the panels in (**B**–**D**) are all based on pdb: 7yf2. (**E**) Conservation of histidine-interacting residues. Parts of a sequence alignment of METTL9 orthologs from diverse organisms are shown, indicating the histidine-interacting residues from (**D**), canonical 7BS motifs and secondary structure elements. METTL9 orthologs from the following organisms are represented in the alignment (with sequence identifiers indicated): *Homo sapiens* (Hs; Q9H1A3-1), *Phytophthora infestans* (Pi; D0NQJ8) *Dictyostelium purpureum* (Dp; XP_003287139.1), *Trypanosoma brucei* (Tb; AAZ11816), *Ostreococcus tauri* (Ot; Q01C52).

Overall, the METTL9 apo structures obtained in the two studies were virtually identical (Figure 3A). In addition to the core 7BS fold, the METTL9 structure also contained an N-terminal, mainly helical segment (residues 37–145) (Figure 3B) which contains several conserved residues involved in binding SAH/SAM or the peptide substrate, e.g., N118, R123 and M126. As with other 7BS MTases, an invariant acidic residue, E174, found in the so-called Motif Post I of the 7BS fold, is involved in SAM coordination and is critical for activity.

The METTL9 recognition motif has two invariant amino acids, i.e., H_−2_ and H_0_. Correspondingly, these two histidines fit snugly into two cavities on the METTL9 surface (Figure 3C). The imidazole ring of H_0_ makes specific contacts with two conserved residues in METTL9, i.e., the main-chain carbonyl group of N210 (which is also involved in SAH coordination) and the side-chain carboxyl group of D213, located within Motif Post II (referred to as “Motif III” by Zhao et al.) [26,27]. H_0_ also interacts with Y295 and L308, which are highly conserved among METTL9 orthologs and lie in β-strands 6 and 7 (β6 and β7), respectively, of the 7BS domain (Figure 3C,D) [26]. These two residues, together with M126 and Y306, another conserved residue in β7, are also involved in the interaction with H_−2_ [26,27]. Importantly, the individual mutation of H_−2_- or H_0_-interacting residues abolished or strongly reduced the enzymatic activity of METTL9 in almost all cases [26,27]. The observed interactions between METTL9 and the more variable residues of the HxH motif, i.e., at the −3, −1 and +1 positions, were more substrate-dependent and appeared less critical. However, the residue at the −1 position (the “x” of the HxH motif) was found to be accommodated in a narrow pocket, which explains why METTL9 activity was diminished or abolished by bulky residues at this position. Also, a hydrogen bond between D300 and the main chain amide of the −3 residue appears critical, as the mutation of D300 abolished METTL9 activity [26,27]. The METTL9 structures also gave important insights into the actual reaction mechanism. The acidic residue D213 was found to form a charge-stabilized hydrogen bond with the N3-atom of H_0_, thus keeping N1 deprotonated and susceptible to engaging in an S_N_2 methylation reaction with SAM (Figure 3D) [26,27].

## 5. Established METTL9 Substrates and Effects of Their Methylation

Although METTL9 recognizes a relatively simple motif (HxH), only five mammalian METTL9 substrates have, in our view, been firmly established so far, namely NDUFB3, S100A9, SLC39A7, DNAJB12, and ARMC6 (summarized in Table 1). All these proteins were found to be robustly methylated by METTL9 in vitro and shown, through dedicated low-throughput analyses, to be methylated in cells. For the first three of these, cellular methylation was also found to be METTL9-dependent (i.e., abolished by METTL9 KO), but for DNAJB12 and ARMC6 this remains to be formally established. Still, we consider these as bona fide METTL9 substrates, since in vitro methylation was demonstrated both with a short peptide and with the full recombinant protein.

The functional effects of METTL9-mediated methylation have been addressed in some depth for three of the established METTL9 substrates—NDUFB3, S100A9, and SLC39A7—and the findings have been summarized below. Histidines are often involved in metal binding/coordination, and, accordingly, the modulation of metal binding to HxH motifs seems to be a common denominator for how METTL9 exerts its functional effects.

### 5.1. The Immunomodulatory and Antibacterial Protein S100A9

Together with its partner S100A8, the S100A9 protein forms the heterodimer denoted calprotectin, which plays an important role both in modulating the immune response and as a direct antimicrobial agent, the latter mediated by sequestering essential trace metals. Interestingly, the histidine residue (H107 in mouse) in S100A9 that is subject to METTL9-mediated methylation is involved in metal coordination, and Daitoku et al. showed that methylation reduced the zinc-binding ability of S100A9 [9].

In a comprehensive study, Cao and co-workers investigated the functional role of the METTL9-mediated methylation of S100A9 during *Staphylococcus aureus* infection in mice [29]. The exposure of neutrophils to *S. aureus* caused a decreased methylation of secreted S100A9, and, during infection, S100A9 from the abscess showed diminished methylation specifically during the acute phase [29]. Moreover, it was observed that S100A9 methylation reduced the ability of recombinant calprotectin (S100A8/S100A9) to inhibit *S. aureus* growth in vitro and in vivo, and, conversely, METTL9 KO mice showed an enhanced ability to fight *S. aureus* infection [29]. Taken together, these results demonstrate that METTL9-mediated methylation is dynamic and diminishes the antibacterial activity of S100A9, suggesting a mechanism for regulating S100A9 by METTL9.

However, while METTL9 appears to influence, and possibly regulate, the antibacterial activity of S100A9 in mice, it is still uncertain whether this also is the case in humans. While the mouse protein contains two tandem HxH motifs, the relevant histidine in human S100A9 is namely in a context (HHH) that is less optimal for METTL9. Thus, while we observed the efficient METTL9-mediated methylation of an S100A9-derived, H107-encompassing peptide from mice, we could not detect a methylation of the corresponding human sequence [10]. Similarly, Daitoku et al. observed that recombinant human S100A9, compared to its mouse counterpart, was inefficiently methylated by METTL9 in vitro [9]. Notably, while all mammalian S100A9 proteins have an HxH motif at the position corresponding to the methylation site in S100A9, many of them have an additional non-conserved C-terminal extension that contains a number of alternating histidines. For example, cow and horse S100A9 have 9–10 additional putative METTL9 sites in this region, and it is tempting to speculate that these sequences are also involved in the METTL9-mediated regulation of S100A9 function.

### 5.2. Mitochondrial Complex I Component NDUFB3

The human mitochondrial complex subunit NDUFB3 is methylated by METTL9 on a stretch of consecutive HxH motifs located very close to the N-terminus. Interestingly, the presence of such HxH motifs is conserved among vertebrates, but the actual number of HxH motifs varies greatly, from only one in some organisms to eight in others [10]. Thus, one may speculate that the METTL9-mediated methylation of these HxH motifs regulates mitochondrial respiration, and that the number of HxHs determines the strength of the regulatory signal. Consequently, the length of the HxH tracts may have evolved to fit the particular requirement of each organism.

In agreement with a possible role for METTL9 in regulating NDUFB3 function, we found that METTL9 knock-out caused a reduction in respiration via Complex I, but not via Complex II [10]. The most prominent effect was observed in the so-called State 3, when respiration fuels ATP synthesis [10]. Importantly, the effect of METTL9 KO could be rescued by the expression of enzymatically active METTL9, but not by an enzyme-dead mutant, indicating that the MTase activity of METTL9 is required for efficient respiration. Reassuringly, an independent study similarly found that the shRNA-mediated knockdown of METTL9 caused decreased respiration in a gastric cancer cell line [28].

As METTL9 has several substrates, one cannot rule out the possibility that the observed reduction in Complex I respiration upon METTL9 KO/KD is not due to the ablation of NDUFB3 methylation. However, this may be clarified by generating cells with a methylation-resistant variant of NDUFB3, e.g., through the mutation of histidines to remove HxH motifs, and then studying the effect of METTL9 KO/KD on respiration; if respiration is unaffected, one may conclude that the effect of METTL9 on respiration is mainly mediated by NDUFB3 methylation.

### 5.3. The Zinc Transporter SLC39A7

Zinc transporter proteins of the SLC30A and SLC39A families frequently contain histidine-rich regions studded with HxH motifs, and several of these transporters have been identified as substrates of METTL9. METTL9 was found to methylate HxH-containing peptides derived from eight zinc transporters (SLC30A1, SLC30A5, SLC30A7, SLC39A4, SLC39A5, SLC39A6, SLC39A7, and SLC39A14) (Table 2) [10], and methylhistidine has been detected in vivo in SLC39A3, SLC39A7, SLC39A9, SLC39A10, and SLC39A14 [8,10,11,30]. Functionally, methylation can modulate zinc binding, illustrated by the reduced zinc affinity of a histidine-methylated SLC39A7-derived peptide and by the observed increase and aggregation of zinc in the cytoplasm of METTL9 KO cells [10,11]. Together, these observations point to a potential role of METTL9 in the regulation of cellular zinc transport.

The zinc transporter that has so far received the most attention as a target of METTL9 is SLC39A7, which transports zinc ions from the ER/Golgi into the cytosol. This protein contains three regions rich in HxH motifs. Two of these regions face the ER lumen and are located at the N-terminus and between transmembrane domains 2 and 3, while the third region is situated between transmembrane domains 3 and 4 and faces the cytosol [31]. MS analyses have identified methylhistidine residues within the ER-facing regions of endogenous or overexpressed SLC39A7 in cell lines, showing that these regions are targeted by METTL9 in vivo [11,30]. In addition, a mutation of the histidines in the loops between transmembrane domains 2 and 3, and between domains 3 and 4, has led to disturbances in zinc levels and distribution in mouse RM-1 cells [11]. Moreover, in mouse mesenchymal stem cells, the methylation of two histidine residues in the N-terminus of SLC39A7 has been shown to limit adipogenic differentiation, which is linked to osteoporosis [32].

Many of the histidine residues within SLC39A7 HxH motifs are involved in zinc binding [33]. Interestingly, the histidines in N-terminal HxH motifs are conserved between animals and plants, but their sequence context differs. In animals, histidines are typically interspaced with small amino acids, whereas in plants the HxH motifs mainly consist of poly-histidine stretches, which are not optimal for METTL9-mediated methylation [33]. This pattern is consistent with the fact that METTL9 is absent in land plants [10,34], reflecting a possible divergence in zinc regulation between plant and animal systems.

**Table 2:** Additional potential substrates of METTL9.

			HxH Methylation		
Substrate	UniProt	Sequence	In Vivo	In Vitro	Reference
AAAS	Q9NRG9	PHSHL	x	x	[10,30]
ACSS2	Q9NR19	GHPHP	x		[30]
AMACR	Q9UHK6	SHSHVHF	x		[30]
AMFR	Q9UKV5	QHNHF	x		[30]
ADISSP	Q9GZN8	SHSHVHF	x		[30]
CCNT1	O60563	NHHHHHNHHSHKHSHS		x	[10]
CCNT2	O60583	SHNHH	x		[8]
CCNT2	O60583	SHKHSHSHS		x	[10]
CEP350	Q5VT06	SHQHV	x		[30]
CNN1	P51911	AHNHHAHN		x	[10]
CPOX	P36551	VHFHR	x		[30]
DNMBP	Q6XZF7	SHPHS	x		[30]
EEF2	P13639	EHAHN	x		[35]
FBXO31	Q5XUX0	FHEHM	x		[8]
HCFC1	P51610	RHSHA	x		[30]
IDH2	P48735	RHAHG		x	[10]
IRF2BP2	Q7Z5L9	LHQHGHS	x		[30]
LMNA	P02545	LHHHHG	x		[8]
LONRF2	Q1L5Z9	GHSHM	x		[30]
MDN1	Q9NU22	GHSHG		x	[10]
MYO18A	Q92614	GHSHG		x	[10]
MYO1D	O94832	KHAHF		x	[10]
NSMF	Q6X4W1	RHPHHHS	x		[30]
NUFIP2	Q7Z417	NHSHNHHHHHHHQ		x	[10]
NUFIP2	Q7Z417	QHHHSHHHPHHHP		x	[10]
PBX1	P40424	MHSHA	x		[30]
PCDHGC4	Q9Y5F7	GHSHG	x		[30]
PDE8B	O95263	SHSHL		x	[10]
PLEC	Q15149	VHSHR	x	x	[10,30]
POLQ	O75417	SHGHR	x		[8]
RAVER1	Q8IY67	GHSHL		x	[10]
RHBDD2	Q6NTF9	CHPHL	x		[30]
S100A9	P06702	GHHHK		x	[9]
SAMM50	Q9Y512	QHVHF	x		[30]
SF3B4	Q15427	GHGHSHPHP		x	[10]
SF3B4	Q15427	GHPHA		x	[10]
SLC25A16	P16260	AHNHH	x		[30]
SLC30A1	Q9Y6M5	GHGHSHGGHGHGHG		x	[10]
SLC30A5	Q8TAD4	DHGHGHSHG		x	[10]
SLC30A7	Q8NEW0	GHGHSHGSGHGHSHS		x	[10]
SLC39A10	Q9ULF5	KHSHHSHG	x		[30]
SLC39A14	Q15043	EHHHGHSHY	x	x	[10,30]
SLC39A3	Q9BRY0	PHGHG	x		[30]
SLC39A4	Q6P5W5	SHSHGGHSHG		x	[10]
SLC39A5	Q6ZMH5	GHSHGHQ		x	[10]
SLC39A6	Q13433	LHHHHHQNHHPHSHS		x	[10]
SLC39A9	Q9NUM3	GHSHK	x		[30]
NHERF1	O14745	FHLHG	x		[30]
STT3B	Q8TCJ2	RHGHH		x	[10]
THSD7B	Q9C0I4	AHHHS		x	[10]
UBR4	Q5T4S7	YHSHK		x	[10]
ZDHHC13	Q8IUH4	NHSHG	x		[30]
ZSWIM8	A7E2V4	AHAHNHL		x	[10]

Listed are HxH-containing sequences, derived from human proteins, that were methylated by recombinant METTL9 in vitro and/or were indicated as likely methylated in datasets of in vivo (in cells or tissues) histidine methylation. Shown are the HxH motif(s) and flanking residues.

## 6. Sequence Specificity and Putative Substrates of METTL9

Although METTL9 seems to be active in most cellular compartments (mitochondria, cytosol, ER, nucleus), it is likely that only a minor subset of the ~2800 ANGST HxH motifs found in the human proteome are methylated by METTL9. Firstly, the METTL9-targeted sequences identified so far are located in flexible disordered sequences, indicating that motifs buried in a protein’s structure will typically not become methylated. Secondly, biochemical investigations demonstrate that, although required, an HxH motif is often not sufficient for methylation to occur. Still, the number of biologically relevant human METTL9 substrates is likely considerably higher than the five currently established, and, to fully understand the biological function of METTL9, it is obviously important to identify its key substrates. Here, we present a catalog of putative METTL9 substrates, encompassing HxH-containing sequences that could be methylated by METTL9 in vitro and/or were found to be methylated in cells, typically through the analysis of high-throughput proteomics data (Table 2). These sequences were also used to generate sequence logos to illustrate METTL9’s sequence specificity (below).

**Figure 4:**
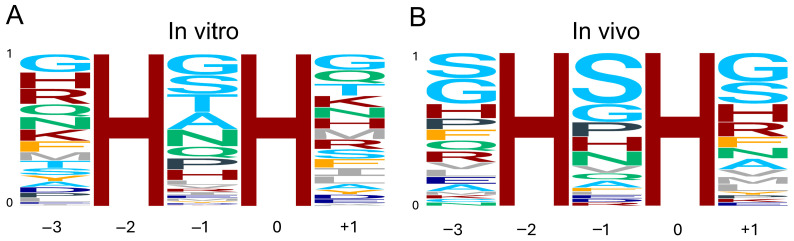
Consensus sequence logo of the METTL9 target motif. (**A**) Logo based on the quantification of in vitro activity of METTL9 on peptide arrays featuring four different peptides with amino acid substitutions at the −3, −1, and +1 positions [10,34]. Note that C and W were not included in this analysis. (**B**) Logo based on frequency of residues in methylhistidine-containing HxH motifs in proteins from cell lines and tissues [8,30,35]. The residues are colored according to side-chain properties: basic (H, K, R), maroon; acidic (D, E), dark blue; small (G, S, T, A), light blue; amide (N, Q), teal; aliphatic (I, L, M, V), gray; aromatic (F, Y), orange; P, charcoal. Logos were generated using the ggseqlogo R package version 0.1 [36].

First, we derived a consensus target sequence logo for METTL9 by analyzing our in vitro METTL9 activity data on peptide arrays featuring four different peptide substrates, each containing a single HxH motif with a substitution at the −3, −1, or +1 position (Figure 4A) [10,34]. At the middle “x” position (−1), there is a clear preference for small residues, with the top five residues G, S, T, A, and N corresponding to the previously mentioned ANGST HxH motif. In addition, residues Q, P, and H appear to be well tolerated at this position (Figure 4A). For both positions flanking the HxH motif, −3 and +1, many residues are permissible. The most favorable residues are G, H, R, Q, N, K, F, and M at the −3 side, and G, Q, T, K, N, H, M, R, and S at the + 1 side. However, it is also clear that most aliphatic, aromatic, and acidic residues are poorly tolerated at the flanks (Figure 4A).

Second, we analyzed the HxH-motifs of peptides identified using MS to contain methylhistidine in cell lines and tissues [8,30,35]. The frequency of each residue at the corresponding position in the dataset was used to generate an “in vivo” METTL9 consensus target sequence logo (Figure 4B). Reassuringly, the substrate preferences obtained from in vivo data are quite similar to those from in vitro analyses. At the −1 position, while S is clearly overrepresented, G, P, H, and N are also frequently observed. Both the in vitro and in vivo analyses indicate that basic residues other than H, as well as aliphatic, aromatic, and acidic residues, are not permissible. However, there are some notable differences, such as the low frequency of A and T at the −1 position in vivo (Figure 4B).

At the flanking positions of the HxH motif, the in vitro and in vivo sequence specificities are also quite consistent. While S is greatly overrepresented, and residues with amide side chains are underrepresented in the in vivo compared to the in vitro data, both analyses clearly indicate that aromatic (except F) as well as acidic and aliphatic residues are not well tolerated at either side (Figure 4A,B).

There are some limitations to the logos we present. For the in vitro logo, the residues C and W were not analyzed for all in vitro peptides and were therefore excluded. However, these residues are rarely found at any of the positions in the in vivo data and are thus likely not very biologically relevant. The in vivo analysis was also complicated by several factors. One is that many of the peptides containing methylhistidine featured several tandem HxH motifs, leading to uncertainties in which residues are in fact at the −3/−1/+1 positions. This may have resulted in, e.g., an overrepresentation of S at the flanking positions of the in vivo data. Additionally, in most cases, we do not strictly know whether methylhistidine-containing HxH motifs in proteins observed in vivo are generated by METTL9 or another histidine methyltransferase, e.g., due to overlapping target sites. Furthermore (as mentioned above; Section 3), sequence and structural features other than the HxH motifs and its flanking residues are also significant determinants for METTL9 activity.

In summary, we conclude that, at the −1 position of the target HxH motif, small amino acids, particularly S and G, along with residues containing amide side, as well as P and H, are preferred for METTL9-mediated methylation. While there are less stringent requirements for the identity of the residues flanking the HxH, it is evident that aromatic residues besides F, as well as acidic and aliphatic residues, are not well tolerated at either side. Despite the abovementioned challenges, and recognizing that the presented logos alone are insufficient to conclusively determine whether an amino acid sequence is a biological METTL9 substrate, we believe they may provide an indication of which proteins are targeted for methylation by METTL9 and thus, together with the presented catalog of putative METTL9 substrates, may be a useful resource for future studies.

It should be mentioned that METTL9 functions not related to protein histidine methylation have also been published. One study reported the *Caenorhabditis elegans* METTL9 ortholog (METL-9) as a DNA methyltransferase introducing N6-methyladenine in genomic DNA [37]. However, we were unable to reproduce these findings and also found that the recombinant *C. elegans* METL-9 protein possessed a robust protein histidine MTase activity [34]. Another recent study found that METTL9 was important for vertebrate neural development but concluded that this function was largely independent of its MTase activity [38]. This was mainly based on a comparison of METTL9 KO cells with cells that expressed a catalytically dead (CD) METTL9 mutant, where the observed changes relative to WT, both regarding gene expression and Golgi fragmentation, were much more dramatic for the KO than for the CD [38]. However, a more disruptive gene targeting strategy was used to generate the KO cells (chromosomal deletions of ~500–1000 bp) than for the CD cells (point mutations). Thus, secondary effects (e.g., on circRNAs or the intron-encoded IGSF6 gene; see below) cannot be excluded, and further studies are, in our view, required to firmly establish an MTase-independent function for METTL9.

## 7. Involvement of METTL9 in Cancer and Other Diseases

METTL9 has been implicated in cancer, though the precise mechanisms remain unclear. Several studies have noted that *METTL9* mRNA expression is elevated in various cancer types, with higher levels associated with a worse prognosis in pancreatic, liver, and gastric cancer [11,28]. Moreover, in both in vitro and in mouse xenograft models, inhibiting METTL9 activity reduced cancer growth and metastatic potential [11,39]. *METTL9* expression levels have also been shown to negatively correlate with immune scores in many different types of cancers, and, correspondingly, METTL9 KO tumors in mouse xenografts contained more immune cells [11].

Several mechanisms have been proposed to explain the cancer-promoting abilities of METTL9. In hepatocellular carcinoma cells, METTL9 was reported to positively regulate the expression of SLC7A11, a protein that inhibits ferroptosis, a form of iron-dependent cell death that influences tumor progression and therapy response [11,40]. Interestingly, SLC7A11 lacks an HxH motif required for methylation by METTL9. However, alterations in intracellular zinc levels are known to influence the regulation of ferroptosis, so an indirect mechanism may exist where the METTL9-mediated methylation of zinc transporters affects SLC7A11 expression and ferroptosis in cancer cells [39,41,42]. A more direct mechanism of METTL9 action has been proposed for metastatic gastric cancer cells, where mitochondria-localized METTL9 promotes the activity of respiratory Complex I, consistent with observations from HEK293 cells [10,28]. Thus, the METTL9-mediated methylation of NDUFB3, or another mitochondrial component, may lead to an increase in intracellular energy production, supporting cancer metastasis.

Interestingly, METTL9 is downregulated in osteoporosis, while SLC39A7 is elevated [32,43]. METTL9 overexpression was reported to suppress bone marrow mesenchymal stem cell adipogenic differentiation and ferroptosis in vitro and in vivo [32]. The effect was ascribed to the methylation of His-45 and His-49 in SLC39A7, since it was abolished by the mutation of these residues. Mechanistically, it was suggested that the METTL9-mediated methylation of SLC39A7 somehow activates the ATF4 transcription factor, which in turn increases levels of SLC7A11, raising intracellular cysteine and glutathione and lowering ferroptosis, thereby inhibiting adipogenesis. METTL9 has also been successfully tested as a therapeutic target for osteoporosis through the injection of an adenovirus overexpressing METTL9 into a mouse model of osteoporosis, which effectively inhibited adipose tissue growth and reduced bone loss [32].

METTL9 is also involved in HIV resistance, as so-called non-progressor HIV patients, who maintain CD4+ T-cell counts within the normal range for several years in the absence of treatment, have a high level of METTL9 expression [44]. Its anti-HIV activity was validated in human cell lines, where the overexpression of METTL9 had an inhibitory effect on HIV production [44]. However, the underlying mechanism remains unclear.

Overall, METTL9 is likely to play a multifaceted role in various diseases through the methylation of distinct subsets of substrates specific to each condition. In the context of cancer, elevated METTL9 expression seems to promote tumor progression and negatively impact patient outcomes, whereas, for HIV progression and osteoporosis, it appears to confer beneficial effects. However, the METTL9 substrates relevant to each condition and their roles remain to be further explored. Understanding these mechanisms could lead to the targeted modulation of METTL9 activity on specific substrates, paving the way for potential therapeutic interventions.

## 8. Functional Significance of a Circular METTL9 RNA (circMETTL9)

**Figure 5:**
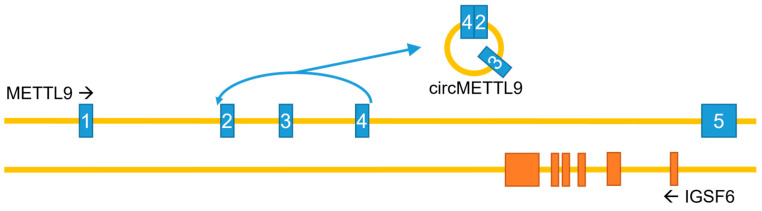
The METTL9 gene—genomic organization and circRNA generation. Note that introns and exons are not drawn to scale, and that the gene IGSF6 in its entirety is encoded by the last intron of the METTL9 gene. METTL9 exons in blue; IGSF6 exons in orange.

In addition to the canonical, protein-coding mRNA, the *METTL9* gene can generate a circular RNA (circMETTL9) through backsplicing, spanning exons 2–4 and the two introns in between (Figure 5). This circMETTL9 appears to be abundant and conserved across several mammalian species. It is highly expressed in muscle tissues, such as mouse hearts and rat vascular smooth muscles, and its levels in pig muscles are high during the prenatal stages and gradually increase as development progresses [45,46,47]. Conversely, the expression of circMETTL9 is downregulated during retinal development in mice, in parallel with the linear *METTL9* transcript, and it is also reduced in the kidneys during fibrosis following unilateral renal ischemia–reperfusion injury [48,49]. Furthermore, circMETTL9 appears to play a role in reproduction, ranking as one of the five most highly expressed circRNAs in pig endometrium, and may also be involved in mouse spermatogenesis [50,51]. CircMETTL9 is also abundantly expressed in astrocytes after traumatic brain injury in rats, and its knockdown increased neurological dysfunction, cognitive impairment, and nerve cell apoptosis induced by this injury. The proposed mechanism involves the direct binding of circMETTL9 to the SND1 protein, leading to an increased expression of cytokines and enhanced neuroinflammation [52].

While the studies mentioned above were all performed in other mammals, more recently, circMETTL9 has also been demonstrated to play an important role in human biology. Upon oxidative stress induced by H_2_O_2_ treatment in neuroblastoma SH-SY5Y cells, circMETTL9 is upregulated, and its knockdown reduces oxidative stress and apoptosis, protecting mitochondrial integrity. In these cells, circMETTL9 appears to act as a protein sponge, binding the CCAR2 protein and positively regulating its expression [53]. Finally, circMETTL9 has been implicated in colorectal cancer (CRC), where it is upregulated in advanced patient tumors, correlating with shorter survival times. Its overexpression promotes the proliferation and migration of colorectal cancer cells in vitro and enhances CRC tumor growth and metastasis in vivo in mouse xenograft models. In the proposed mechanism, circMETTL9 interacts with the miR-551b-5p microRNA, acting like a miRNA sponge to control the expression of the CDK6 oncogene [54]. In summary, circMETTL9 appears to be involved in many biological processes. Therefore, especially in the context of gene editing and RNA silencing, both METTL9 protein function and the circular RNA should be kept in mind, since perturbing the locus may alter the functions of both products.

## 9. Conclusions and Future Perspectives

Only five years ago, virtually no information on METTL9 could be found in the scientific literature. In addition, METTL9 showed no particular sequence similarity to other human 7BS MTases, making it difficult to predict whether it would methylate proteins, nucleic acids, or small molecules. As summarized in this review, a large and rapidly growing body of work on METTL9 now defines its biochemical activity and key structure–function relationships. Several in vivo substrates of METTL9 have been identified, and, for some of these, a functional role of METTL9 methylation has been established. Still, several important questions remain, such as determining whether METTL9 activity is subject to regulation, identifying the complete set of its biologically significant substrates, and defining the specific cellular and physiological processes affected by METTL9-dependent histidine methylation.

## Figures and Tables

**Table 1:** Established METTL9 in vivo substrates and effects of methylation.

Substrate	UniProt	Sequence(s) ^a^	Residues	Effect	Reference
Complex I subunit β3 (NDUFB3)	Q02365 O43676	AHG**H**G**H**E**H**G (*Bt*) AHEHGHEHG	2–10 2–10	Increased Complex I mediated respiration	[5,10,28]
Armadillo repeat-containing protein 6 (ARMC6)	Q6NXE6	GHA**H**NHA	260–266	Unknown	[10]
DnaJ homolog B12 (DNAJB12)	Q9NXW2	RHGHGHG	182–188	Unknown	[10]
Protein S100A9	P31725	GHGHS**H**G (*Mm*)	102–108	Decreased metal binding and antibacterial activity	[6,9,10,29]
Zinc transporter SLC39A7	Q92504 Q31125	GHGHDHEHSHG GHGHS**H**GHG (*Mm*)	105–115 251–259	Decreased Zn^2+^ binding	[8,10,11]

^a^ The shown sequences were found to be histidine-methylated in cells, and, if known, the actual methylated histidines are shown in bold and underlined. The sequences represent human proteins, except when indicated otherwise. *Bt*, *Bos taurus* (cow); *Mm*, *Mus musculus* (mouse).

## Data Availability

No new data were created or analyzed in this study.

## Abbreviations

The following abbreviations are used in this manuscript:

1MH	1-methylhistidine
3MH	3-methylhistidine
MTase	Methyltransferase
SAH	*S*-adenosylhomocysteine
SAM	*S*-adenosylmethionine
7BS	Seven-β-strand
